# An investigation of the relationships between suicidal ideation, psychache, and meaning in life using network analysis

**DOI:** 10.1186/s12888-023-04700-4

**Published:** 2023-04-17

**Authors:** Yijun Li, Zhihua Guo, Wenqing Tian, Xiuchao Wang, Weijia Dou, Yanfeng Chen, Shen Huang, Shengdong Ni, Hui Wang, Chaoxian Wang, Xufeng Liu, Xia Zhu, Shengjun Wu

**Affiliations:** 1 Department of Military Medical Psychology, Air Force Medical University, Xi’an, China; 2 Department of Gastroenterology, Tangdu Hospital, Air Force Medical University, Xi’an, China; 3 No.66168 Hospital, Beijing, China; 4 Xi’an Research Institute of High Technology, Xi’an, China; 5 School of Construction Machinery, Chang’an University, Xi’an, China; 6 The troops of Peoples’s Liberation Army, Xi’an, China; 7 Air Force Medical University, No. 169 West Changle Road, Xi’an, Shaanxi Province, 710032 China

**Keywords:** Meaning in life, Network analysis, Prevention, Psychache, Suicidal ideation

## Abstract

**Background:**

Previous studies have investigated the relationships between psychache or meaning in life and suicidal ideation based on sum score of corresponding scale. However, this practice has hampered the fine-grained understanding of their relationships. This network analysis study aimed to conduct a dimension-level analysis of these constructs and the relationships among them in a joint framework, and identify potential intervention targets to address suicidal ideation.

**Methods:**

Suicidal ideation, psychache, and meaning in life were measured using self-rating scales among 738 adults. A network of suicidal ideation, psychache, and meaning in life was constructed to investigate the connections between dimensions and calculate the expected influence and bridge expected influence of each node.

**Results:**

“Psychache” was positively linked to “sleep” and “despair”, while “presence of meaning in life” had negative associations with “psychache”, “despair”, and “pessimism”. The most important central nodes were “sleep” and “despair”, and the critical bridge nodes were “presence of meaning in life” and “psychache”.

**Conclusion:**

These preliminary findings uncover the pathological pathways underlying the relationships between psychache, meaning in life, and suicidal ideation. The central nodes and bridge nodes identified may be potential targets for effectively preventing and intervening against the development and maintenance of suicidal ideation.

**Supplementary Information:**

The online version contains supplementary material available at 10.1186/s12888-023-04700-4.

## Background

Suicidal ideation is defined as thoughts ranging from a vague idea of committing suicide to a specific suicide plan [1]. Some researchers have claimed that suicidal ideation is an important phase in the process of suicide, usually preceding suicide attempts and completed suicide [2]. Suicidal ideation is reported to be closely correlated with subsequent suicide attempts and completed suicide [3, 4]. An 18-month follow-up survey has shown that persistent suicidal ideation is positively associated with risk of completing suicide [5]. In addition, results of a nationwide study conducted in Finland [6], showed that 22% of those who had committed suicide had expressed suicidal ideation within 28 days before death during their last visit with a health care professional. Furthermore, a meta-analysis has also revealed that those who had reported or expressed suicidal ideation had a greater risk for completed suicide compared with those who had not, including both psychiatric and non-psychiatric populations [7]. Therefore, many investigators believe that suicidal ideation is among the most important risk factors for completed suicide and increased suicide mortality [7–11].

Given that suicide is a leading cause of death worldwide [12, 13], coupled with suicidal ideation being a major precursor to completed suicide, substantial efforts should be undertaken to investigate primary contributors to the development and maintenance of suicidal ideation. In other words, it is imperative to identify the pathogenesis relevant to suicidal ideation (e.g., pathological pathways, risk factors, and protective factors) to increase the effectiveness of suicide prevention and intervention.

Several psychological variables have been linked to suicidal ideation, and psychache is a critical psychological variable that contributes to suicidal ideation. According to Shneidman’s theory of suicide [14], psychache is defined as hurt, anguish, soreness, aching, and psychological pain in the mind; Shneidman proposed that when an individual experiencing psychache deems the pain unbearable, suicidal ideation to escape from it through suicide occurs. Support for psychache as the cause of suicidal ideation is strong, regardless of whether it occurs in the context of a mood disorder or in the absence of a mental disorder [15]. Previous studies have shown that psychache is closely related to, and is a prominent predictor of suicidal ideation [15–23]. Furthermore, a recent longitudinal study has revealed that psychache and suicidal ideation are reciprocally inter-related over time [24]. Altogether, psychache is a core psychological construct for understanding suicidal ideation and suicide, and studies on the relationship between them may help develop effective interventions for suicidal ideation.

Meaning in life has also been shown to be an important protective factor against suicidal ideation [25–27]. Meaning in life is defined as the sense made of, and significance felt regarding, the nature of one’s being and existence and is regarded as a cornerstone of well-being [28, 29]. Meaning in life is also positively related to mental health and inversely associated with negative emotions [25]. For instance, a previous study has found that participants with low meaning in life had higher levels of suicidal ideation than those with high meaning in life [25]. Meaning in life also decreases the risk of non-suicidal self-injury, suicidal thoughts and behaviors [30]. Overall, meaning in life has a close relationship with suicidal ideation, and the evidence indicates that it plays a major protective role against it. Furthermore, a close relationship between loss of meaning in life and intense psychache has also been shown in some studies [31], and greater meaning in life is inversely related to psychache [32].

Previous studies have used sum scores of various scales to examine the relationship between suicidal ideation and psychache [16, 17, 20], and relationships between suicidal ideation and meaning in life [25–27]. However, this approach may ignore the fine-grained relationships between the dimensions of these psychological constructs and obscure the degree of significance of different dimensions [33, 34]. For example, meaning in life is a multi-dimensional construct. Some investigators hold that it contains two dimensions: the presence of meaning in life and a search for meaning in life [26, 28, 35]. Similarly, suicidal ideation and psychache are also multidimensional constructs. To date, research based on a holistic perspective has hampered progress in this area such as identifying pathological pathways between psychopathological constructs which might suggest appropriate targets for effective therapies [36]. However, performing analysis of individual dimensions offers a promising way forward. Furthermore, previous studies of suicidal ideation, meaning in life, and psychache have rarely been investigated in a joint framework, although they are closely related, and is an additional shortcoming of previous research. To better understand the pathological pathways between the three constructs, especially between psychache and suicidal ideation, and between meaning in life and suicidal ideation, and to identify potential factors for effective and targeted interventions for suicidal ideation, studies that examine the relationships among the three constructs at a fine-grained level are needed.

Network model (i.e., network analysis) is an emerging and promising data-driven approach to psychopathology, and is able to reveal relationships between dimensions [37, 38]. Network analysis visualizes psychopathological constructs as a network consisting of nodes (dimensions or factors) and node-to-node edges (correlations between individual dimensions/factors) [39, 40]. The mental disorder network may include different associated constructs (also known as communities) and their corresponding dimensions. Network theory asserts that active interaction and mutual reinforcement of dimension nodes cause the emergence of the psychopathological construct network, rather than passively regarding individual dimensions as reflections of a latent psychopathological variable [37, 38, 41].

Utilizing network analysis to investigate psychopathological constructs has some advantages and scientific implications. The approach permits analysis of individual dimensions of constructs at a fine-grained level, overcoming the shortcomings of previous studies that evaluate relations between constructs in the single-ensemble form. It involves evaluating the importance of edges that may uncover pathways among psychopathological constructs. Moreover, network analysis can provide a predictability index for each node to assess the controllability of the node in the whole network [42]. It also provides centrality indices to identify important central nodes that activate all other nodes and exert great influence on the overall network [38, 43, 44]. In addition, it is also able to assess bridge centrality indices to determine dominant bridge nodes that are critical to maintain the co-occurrence of psychopathological constructs and facilitate the impact of one construct on others [40, 45–47]. However, to our knowledge, no study has investigated the relationships among suicidal ideation, psychache, and meaning in life via network analysis, not to mention using a joint framework.

To address this research gap, we constructed a network structure of suicidal ideation, psychache, and meaning in life and assessed the characteristics of the network. The aims of the study were: (1) to shed light on the pathological pathways between psychache and suicidal ideation, and between meaning in life and suicidal ideation; (2) to determine the critical central nodes that maintain the whole network; (3) to identify the predominant bridge nodes that connect different constructs; and (4) provide preliminary suggestions for the targeted prevention of, and interventions for suicidal ideation. Given that no study has investigated the fine relationships among the three constructs, our study is largely exploratory and extends the research field.

## Methods

### Participants and ethical approval

This study used an online survey hosted on the Wenjuanxing platform (www.wjx.cn). A total of 800 adults aged 18 years and older were recruited through convenience sampling based on WeChat from 2 to 2022 to 9 June 2022. Participants were eligible for inclusion if they (1) were healthy based on self-report; (2) with no self-reported history of neurological or psychiatric illnesses; and (3) consented to participate in the study. Participants gave their electronic informed consent after being informed about the purpose and nature of the research and the rights and obligations of the researcher and participants. The anonymity of the study was emphasized to encourage honest responses. Finally, participants completed three self-report scales (see below). Of course, participants could discontinue the survey at any time. To control data quality, 62 surveys were excluded because they met the following criteria: (1) the time used to complete the survey was < 100 s suggesting it was completed without thinking about each question, and (2) concealment dimension score was ≥ 4 on the Self-rating Idea of Suicide Scale. Our study was approved by the Ethics Committee of Xijing Hospital, Air Force Medical University.

### Measures

#### Self-rating scale for suicidal ideation (SIOSS)

The 26-item SIOSS was used to measure suicidal ideation [48, 49]. It includes four dimensions: despair, optimism, sleep, and concealment, and items are answered with a “yes” or “no”. If the concealment dimension score is ≥ 4, the measurement is considered unreliable. If the total score of despair, optimism and sleep dimensions is ≥ 12, the participant is considered to have suicidal ideation, with higher scores indicative of stronger suicidal ideation. The optimism dimension represents the opposite connotations because only the answers indicating negative meanings score. For the convenience of understanding, this paper used pessimism dimension to re-label the optimism dimension. In this study, Cronbach’s α coefficient of the scale was 0.77.

#### The psychache scale (PAS)

The Chinese version of PAS, a single-dimension questionnaire with a total of 13 items, was used to assess psychache [50, 51]. Each item is rated on a 5-point Likert-type scale ranging from 1 = *never* to 5 = *always* or 1 = *strongly disagree* to 5 = *strongly agree*. The scale is used to evaluate the introspective experience of negative emotions such as guilt, despair, loss, and fear. The higher the total score of the scale, the greater the psychache perceived by the individual. The Cronbach’s α coefficient of the scale was 0.96.

#### Chinese meaning in Life Questionnaire (C-MLQ)

MLQ was translated into Chinese and used to evaluate each participant’s meaning in life and his/her pursuit of it [28, 52]. It contains two dimensions: the presence of meaning in life and search for meaning in life. The scale has a total of 10 items that are rated on a 7-point Likert-type scale ranging from 1 = *absolutely untrue* to 7 = *absolutely true*. The higher the score, the higher the individual’s meaning in life. The Cronbach’s coefficient α of the total scale was 0.86 in our sample.

### Network analysis

The network was estimated via Gaussian graphical model (GGM), which is an undirected network [53]. In the model, each dimension of the scales (SIOSS, PAS, C-MLQ) was regarded as a node, and the partial correlation between two nodes after statistically controlling for any influence from other nodes was regarded as an edge. The estimation of GGM was based on nonparametric Spearman correlations [54]. The least absolute shrinkage and selection operator (LASSO) and Extended Bayesian Information Criterion (EBIC) were used to regularize the GGM [55, 56]. In this process, the edge with small partial correlation was set to zero, thus edges were shrunk and the symptom network was sparser and easier to interpret [54, 55]. Meanwhile, setting the tuning parameter to 0.5 balances the sensitivity and specificity of extracting true edges well [54]. R-package *qgraph* was used to construct and visualize the network in this part [57].

The expected influence (EI) of each node was calculated as the centrality index using R-package *qgraph* [57]. The EI value indicates the importance of the node for the entire network, and the higher the EI, the more influential the node. Bridge expected influence (BEI) was calculated as the bridge centrality indicator by R-package *networktools* [45]. A higher BEI value means a higher risk of contagion from the current community to other communities. In the present network, nodes were divided into three communities prior to analysis: suicidal ideation (three nodes), meaning in life (two nodes) and psychache (one node). Moreover, the R-package *mgm* was used to calculate the predictability of each node, which is an indicator reflecting the degree to which the variance of a node can be explained by all of its neighboring nodes and the controllability of the network model [42].

The accuracy and stability of the network were evaluated via R-package *Bootnet* [39]. First, the accuracy of edge weights was examined with the bootstrapped 95% confidence interval (CI) based on 1000 bootstrap samples. A narrower CI indicates the estimation of the edge weights is more accurate [58]. Second, the correlation stability (CS) coefficient calculated by a case-dropping bootstrap approach (1000 bootstrap samples) was used to evaluate the stability of the estimations of EI and BEI. A value greater than 0.5 indicates strong stability [39]. Third, bootstrapped difference tests (1000 bootstrap samples) were conducted for testing the differences of edge weights, EIs, and BEIs.

## Results

The mean age of the 738 participants was 23.51 ± 3.93 years (mean ± SD, range = 18–46 years), and the majority were aged 30 years and younger (93%), with only 7% aged older than 30. Most participants were male (n = 700, 94.8%). Thirty-four participants (4.6%) met the criteria to screen individuals with suicidal ideation. The mean scores, standard deviations, EI (raw values), BEI (raw values), and predictability for each variable are shown in Table 1.

**Table 1:** The mean scores, standard deviation, EI, BEI, and predictability for each variable

Variable	M ± SD	EI	BEI	Pre
Despair	1.38 ± 2.51	0.55	0.10	0.68
Pessimism	0.21 ± 0.62	0.09	–0.16	0.86
Sleep	0.70 ± 1.07	0.68	0.29	0.80
Psychache	16.45 ± 6.43	0.38	0.38	0.79
MLQ-P	29.31 ± 5.41	–0.41	–0.69	0.78
MLQ-S	24.80 ± 8.12	0.42	0.14	0.97

Note: M, mean; SD, standard deviation; EI, expected influence; BEI, bridge expected influence; Pre, predictability; MLQ-P, presence of meaning in life; MLQ-S, search for meaning in life

Blue edges represent positive relations, whereas red edges represent negative relations. The thickness of an edge indicates strength of the relationship. The weights of edges are provided in Supplementary Table 1. The ring around each node depicts its predictability. MLQ-P, presence of meaning in life; MLQ-S, search for meaning in life

**Fig. 1:**
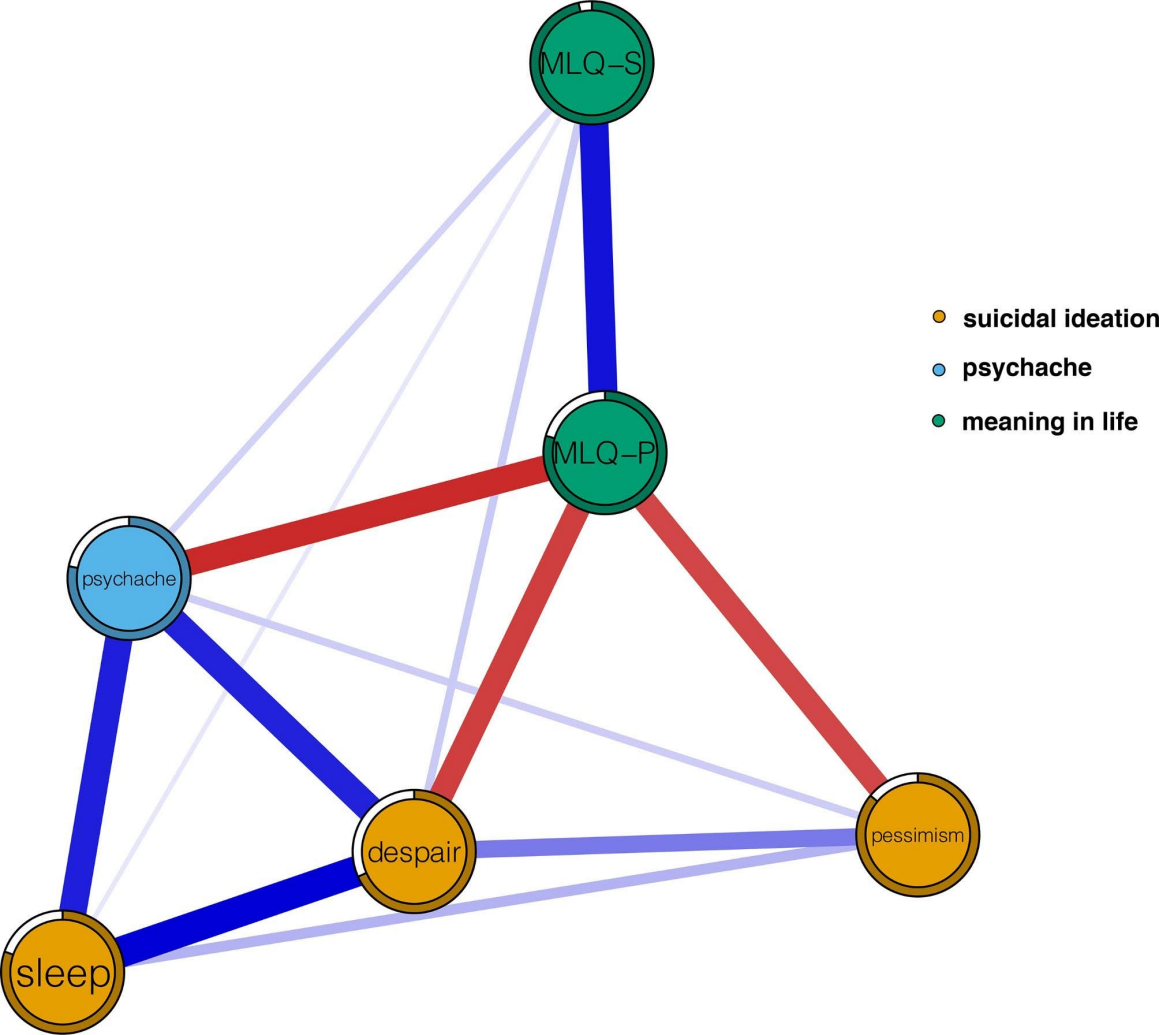
The network structure of suicidal ideation, psychache and meaning life

The final network is shown in Fig. 1, and several characteristics were apparent. First, there were 13 (86.67%) non-zero edges of 15 possible edges, including three negative and 10 positive edges. Second, the two strongest edges in the network structure appeared within the “suicidal ideation” and “meaning in life” communities. In the “suicidal ideation” community, the strongest edge was between “sleep” and “despair” (weight = 0.30). Within the “meaning in life” community, the second strongest edge (weight = 0.28) linked “presence of meaning in life” and “search for meaning in life”. Although relatively weak, some cross-community edges were also found. “Psychache” was positively linked to “sleep” and “despair”, with both edge weights equaling 0.26. “Presence of meaning in life” had negative associations with “psychache”, “despair”, and “pessimism” (weight = − 0.25, − 0.22, − 0.22, respectively). All edge weights of the present network can be seen in Supplementary Table 1. The bootstrapped 95% CI was narrow, suggesting that the estimation of edge weights was accurate and stable (Supplementary Fig. 1). The bootstrapped difference test for edge weights is shown in Supplementary Fig. 2. Third, predictability for each node was represented by a ring around it, and values ranged from 68 to 97%. The average node predictability was 81% (see Table 1), indicating 81% of the variance of the nodes could be explained by their neighboring nodes.

**Fig. 2:**
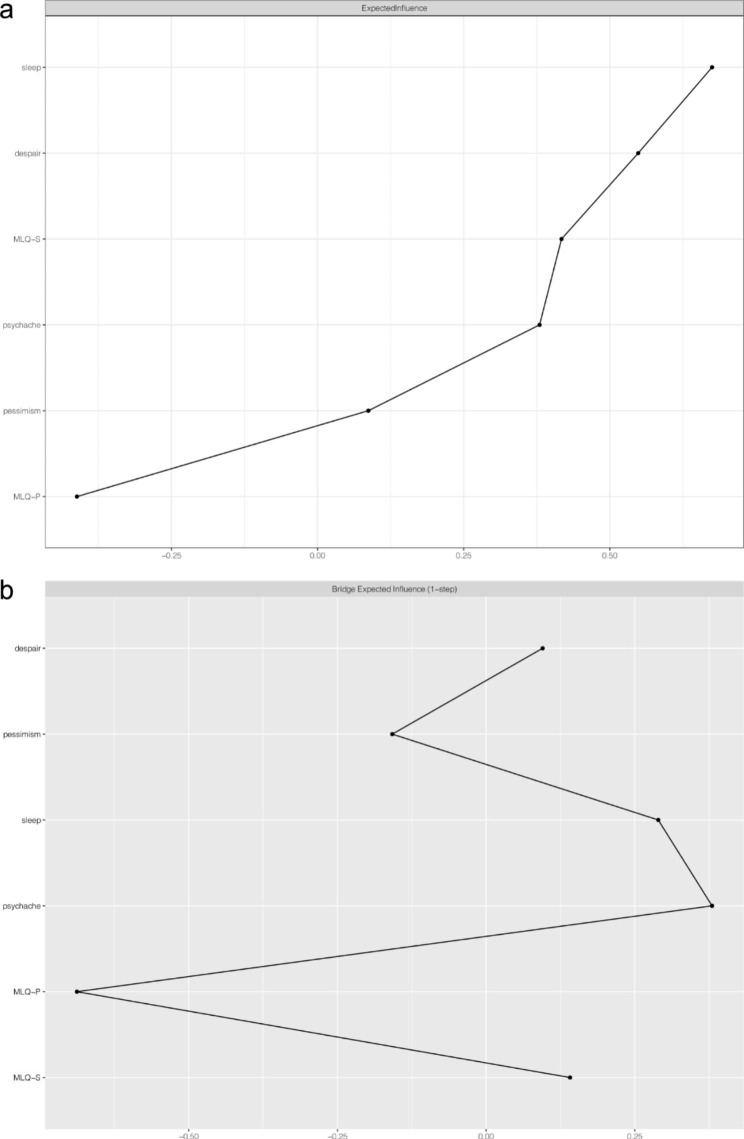
The expected influence and bridge expected influence of each node in the present network (raw value) (a) Expected influence. (b) Bridge expected influence. MLQ-P, presence of meaning in life; MLQ-S, search for meaning in life

Node EI values were calculated to assess their relative importance in the current network (see Fig. 2a; Table 1). The nodes “sleep” (EI = 0.67) and “despair” (EI = 0.55) had the highest EI values, making them the most important central nodes. The CS coefficient for EI was 0.75, indicating the estimation of node EI had a good level of stability (see Supplementary Fig. 3). The result of the bootstrapped difference test for node EI is shown in Supplementary Fig. 4.

BEI values for each node are shown in Fig. 2b. In the “meaning of life” community, a critical bridge node was “presence of meaning in life” which exhibited the highest BEI with a value of − 0.69. Another critical bridge node was “psychache” (BEI = 0.38). The CS coefficient for BEI was 0.75, exceeding the preferable threshold of 0.5, signifying the estimation of BEI was adequately stable (see Supplementary Fig. 5). Supplementary Fig. 6 shows the result of the bootstrapped difference test for node BEI.

## Discussion

While existing studies have demonstrated that psychache and meaning in life are associated with suicidal ideation [16, 27], the present study is the first to explore the fine-grained relationships among them via network analysis. Specifically, we examined the dimensional-level relationships between psychache and suicidal ideation, and between meaning in life and suicidal ideation. Our findings may facilitate our understanding of the specific pathological pathways underlying the close relationships between psychache, meaning in life and suicidal ideation. The study also identified the critical central nodes and bridge nodes that play important roles in developing and maintaining suicidal ideation, which suggest potential targets for prevention and intervention strategies to address suicidal ideation.

The results showed that the two strongest edges existed within the community. These findings are similar to previous network analysis studies that revealed the strongest edges existed within the community when examining network structures composed of different communities [40, 46, 59–61]. In addition to within-community edges, strong cross-community edges were also found, of which the edges between suicidal ideation and psychache or meaning in life were of most interest to us. The results revealed that “psychache” was positively associated with the “despair” and “sleep” dimensions of suicidal ideation, which may be the mechanisms underlying psychache as an important risk factor for developing suicidal ideation [17, 20, 23, 24]. This result is consistent with studies that suggest that considering suicide is a solution to escaping from unbearable psychache associated with feeling of despair caused by unfulfilled psychological needs such as security and accomplishment [17, 62]. As for the association between “psychache” and “sleep”, previous studies have shown that psychache aggravates the adverse effects of sleep disturbance on suicidal ideation and suicide attempts [63]. Since no studies have investigated the network structure of psychache and suicidal ideation, the current study provides a preliminary insight into their relationships and further studies are warranted.

We also found that “presence of meaning in life” had strong negative connections with the “despair” and “pessimism” dimensions of suicidal ideation, which is consistent with previously reported findings that meaning in life has a positive effect on preventing suicidal ideation and suicide [25–27]. Previous research has also suggested that individuals confused about meaning in their life are highly likely to experience despair and think about suicide [64–66]. One study has also proposed that creating moments of meaning in life can reduce despair [67]. Our finding is also consistent with previous studies that have shown the presence of meaning in life contributes to decreased suicidal ideation and is a protective factor against suicide [26, 27]. Furthermore, a multinational study found that meaning in life was positively related with optimism via multivariate analysis [68], which is also consistent with our finding of a negative relationship between “presence of meaning in life” and “pessimism”. Additionally, our results showed that “presence of meaning in life” was negatively related to “psychache”, which accords with the results of previous studies [31, 32]. It demonstrates another potential mechanism for the protective role of meaning in life in decreasing suicidal ideation: “presence of meaning in life” indirectly decreases suicidal ideation via its negative association with psychache — a susceptibility factor for suicidal ideation. The average predictability of the whole network was 81%, implying that the current network is more likely to be self-determined [42, 59].

The expected influence result showed that the dominant central nodes were the “sleep” and “despair” dimensions of suicidal ideation, meaning that “sleep” and “despair” exert great influence on the network. This is partly consistent with a previous study that showed sleep symptoms were central within the suicidal ideation networks of both males and females [69]. However, few studies have investigated suicidal ideation at a dimensional level, and most network studies regard suicidal ideation as a symptom of depression only [70, 71], and are therefore, not directly comparable with our study. Our finding that “sleep” and “despair” are central nodes is largely exploratory and is worth further consideration and investigation. Additionally, we calculated the bridge expected influence for each node to identify critical bridge nodes, and the results indicated that “presence of meaning in life” and “psychache” were bridge nodes. Our finding that “presence of meaning in life” was negatively linked to “despair”, “pessimism”, and “psychache”, indicates that presence of meaning in life prevents the despair and pessimism dimensions of suicidal ideation, and decreases psychache to reduce suicidal ideation. These findings are also consistent with previous studies [32, 67, 68]. However, we found “psychache” was the opposite case in the current study, indicating that individuals with psychache are susceptible to suicidal ideation.

The current study has important theoretical and clinical implications. Regarding the theoretical implications, these findings provide preliminary insights into the potential pathological pathways linking between psychache, meaning in life and suicidal ideation, furthering our understanding of the mechanisms underlying their relationships. Our findings are of great importance to figure out specific roles played by different dimensions of meaning in life or psychache in the development and maintenance of dimensions of suicidal ideation. In detail, this study suggests that the positive relationships between “psychache” and “sleep” or “despair” may explain how psychache contributes to suicidal ideation. Moreover, our study further suggests a possible pathway by which the meaning in life may reduce suicidal ideation, i.e., through the negative effect of “presence of meaning in life” on “despair” or “pessimism” dimensions of suicidal ideation. Additionally, the negative association between “presence of meaning in life” and “psychache” also indirectly accounts for the protective effect of meaning in life on decreasing suicidal ideation. Regarding the clinical implications, these findings provide an important reference for developing the strategies of clinical prevention and intervention for coping with suicidal ideation. Central nodes play an important role in activating other symptoms and developing and maintaining mental disorders, and exert a great influence on the whole psychopathological network [38, 72, 73]. Therefore, central nodes are regarded as promising targets for effective intervention [40, 46, 61, 74]. In our study, “sleep” and “despair” played the most important roles in the activation and maintenance of the network of suicidal ideation, psychache, and meaning in life, and hence, interventions targeting these two dimensions may effectively attenuate suicidal ideation and psychache. Similarly, bridge nodes are critical for the co-occurrence of psychopathological constructs and facilitate the impacts of one construct on others; therefore, bridge nodes are also considered targets for prevention and intervention [40, 44, 45, 47, 75]. In our study, “presence of meaning in life” and “psychache” were both identified as crucial bridge nodes for developing and maintaining suicidal ideation, indicating they might be promising targets for intervention. Controlling the adverse influence of “psychache”, as well as enhancing the protective effect of “presence of meaning in life” on suicidal ideation might increase the effectiveness of prevention and intervention strategies to mitigate suicidal ideation and suicide risk.

Although these findings are important, several limitations should be noted. First, due to the cross-sectional design of our study, we cannot make any inferences regarding causality between any of the constructs examined. Longitudinal or experimental studies are needed to examine the causal relationships among the constructs. Second, the study sample was mainly young male adults, which led to the uneven distribution of the age and sex, weakening the representativeness of the participants and limiting generalizability of our findings. Hence, future studies are required to include more females and other age groups in the analysis and the applicability of our results to other populations also requires replication in other samples. Third, the data was collected via self-report scales. Therefore, the results may have been influenced by recall bias [61, 76], and our findings should be interpreted with caution. Fourth, we used only one scale to measure each construct, and we may not have included all dimensions of those constructs. The current study only provided limited insight into their relationships, and future research using other scales that include other dimensions of the constructs are needed to comprehensively investigate how psychache and meaning in life develop and maintain suicidal ideation. Fifth, the self-report nature of our study may also hamper the validity of exclusion criteria. For example, individuals with depression can be included if they self-reported them as non-psychiatric; and the nodes “sleep” and “despair” happened to be symptoms of a current depressive episode, which is a reasonable mediator of suicidal ideation. This may add confounding factors to our study and undermine the quality and validity of our findings, which reminds us to interpret the findings cautiously. Sixth, although online recruitment and survey benefited us a lot, such as obtaining a large amount of data in a short time, saving a lot of manpower and resources, and reducing the effect of social approval due to its anonymity, it also introduced some challenges. For example, respondents are clustered in the younger population because the younger adults use the Internet and WeChat more often, which can also result in the sample of studies having risk of bias. Finally, although we identified some central and bridge nodes that might be potential targets for preventing suicidal ideation and intervening against it, the effectiveness of any such treatments requires thorough investigation and evaluation.

## Conclusion

This study presents the first application of network analysis to investigate the relationships between suicidal ideation, psychache, and meaning in life in a joint framework. Our findings confirm the close relationship between both psychache and meaning in life, and suicidal ideation (the former being a risk factor and the latter a protective factor) at a dimensional level. The identification of “sleep” and “despair” as predominant central nodes and “presence of meaning in life” and “psychache” as critical bridge nodes have important clinical applications insofar as these nodes may be potential targets for effectively preventing and intervening against suicidal ideation.

## Electronic supplementary material

Below is the link to the electronic supplementary material.

Supplementary Material 1 Supplementary Table and Figures

## Data Availability

The datasets used and/or analysed during the current study are available from the corresponding author on reasonable request.

## List of abbreviations

SIOSS: Self-Rating Scale for Suicidal Ideation
PAS: The Psychache Scale
C-MLQ: Chinese Meaning in Life Questionnaire
GGM: Gaussian graphical model
LASSO: Least absolute shrinkage and selection operator
EBIC: Extended Bayesian Information Criterion
EI: Expected influence
BEI: Bridge expected influence
Pre: Predictability
MLQ-P: Presence of meaning in life
MLQ-S: Search for meaning in life
