# Supplementary material for: An investigation of the relationships between suicidal ideation, psychache, and meaning in life using network analysis
Source: BMC Psychiatry. 2023 Apr 17;23:257. doi: 10.1186/s12888-023-04700-4 (PMC10111716; doi:10.1186/s12888-023-04700-4)
Supplement: Supplementary file 1 — Supplementary Material 1 Supplementary Table and Figures [file 12888_2023_4700_MOESM1_ESM.docx]

Supplementary materials

Supplementary Table 1 All edges weights within the network

|  | despair | pessimism | sleep | psychache | MLQ-P | MLQ-S |
| --- | --- | --- | --- | --- | --- | --- |
| despair | 0 | 0.16 | 0.30 | 0.26 | -0.22 | 0.06 |
| pessimism | 0.16 | 0 | 0.09 | 0.06 | -0.22 | 0 |
| sleep | 0.30 | 0.09 | 0 | 0.26 | 0 | 0.03 |
| psychache | 0.26 | 0.06 | 0.26 | 0 | -0.25 | 0.05 |
| MLQ-P | -0.22 | -0.22 | 0 | -0.25 | 0 | 0.28 |
| MLQ-S | 0.06 | 0 | 0.03 | 0.05 | 0.28 | 0 |

*Note*: MLQ-P, presence of meaning in life; MLQ-S, search for meaning in life.

Supplementary Figure 1 Accuracy of edge weights in the network

*Note*: The red line depicts the sample edge weights and the gray bar depicts the bootstrapped confidence interval.

Supplementary Figure 2 Bootstrapped difference test for edge weights in the network

*Note*: Gray boxes indicate edge weights that do not differ significantly from one another, while black boxes indicate edge weights that do differ significantly. Blue and red boxes on the diagonal correspond to edge weights with positive and negative correlations, respectively.

Supplementary Figure 3 Stability of node expected influences in the network

*Note*: The red bar represents the average correlation between node expected influences in the full sample and subsample with the red area depicting the 2.5th quantile to the 97.5th quantile.

Supplementary Figure 4 Bootstrapped difference test for node expected influences in the network

*Note*: Gray boxes indicate node expected influences that do not differ significantly from one another, while black boxes indicate node expected influences that do differ significantly. The numbers in the white boxes (i.e., diagonal line) represent the values of node expected influences.

Supplementary Figure 5 Stability of node bridge expected influences in the network

Note: The red bar represents the average correlation between node bridge expected influences in the full sample and subsample with the red area depicting the 2.5th quantile to the 97.5th quantile.

Supplementary Figure 6 Bootstrapped difference test for node bridge expected influences in the network

Note: Gray boxes indicate node bridge expected influences that do not differ significantly from one another, while black boxes indicate node bridge expected influences that do differ significantly. The numbers in the white boxes (i.e., diagonal line) represent the values of node bridge expected influences.
